# Gut and Urinary Microbiota in Cats with Kidney Stones

**DOI:** 10.3390/microorganisms12061098

**Published:** 2024-05-29

**Authors:** Patrick Joubran, Françoise A. Roux, Matteo Serino, Jack-Yves Deschamps

**Affiliations:** 1 Nutrition, PathoPhysiology and Pharmacology (NP3) Unit, Oniris VetAgro Bio, Nantes-Atlantic College of Veterinary Medicine, Food Science and Engineering, La Chantrerie, CEDEX 03, 44 307 Nantes, France; patrick.joubran@oniris-nantes.fr (P.J.); francoise.roux@oniris-nantes.fr (F.A.R.); 2 Emergency and Critical Care Unit, Oniris VetAgro Bio, Nantes-Atlantic College of Veterinary Medicine, Food Science and Engineering, La Chantrerie, CEDEX 03, 44 307 Nantes, France; 3 IRSD, Institut de Recherche en Santé Digestive, Institut National de la Santé et de la Recherche Médicale (INSERM) U1220, Institut National de Recherche pour l’Agriculture, l’Alimentation et l’Environnement (INRAE), Ecole Nationale Vétérinaire de Toulouse (ENVT), Université de Toulouse III-Paul Sabatier (UPS), CS 60039, 31 024 Toulouse, France

**Keywords:** kidney stones, calcium oxalate, gut-kidney axis, urinary microbiota, feline urobiome

## Abstract

Upper urinary tract urolithiasis is an emerging disease in cats, with 98% of kidney stones composed of calcium oxalate. In humans, disturbances in the intestinal and urinary microbiota are suspected to contribute to the formation of calcium oxalate stones. We hypothesized that similar mechanisms may be at play in cats. This study examines the intestinal and urinary microbiota of nine cats with kidney stones compared to nine healthy cats before, during, and after treatment with the antibiotic cefovecin, a cephalosporin. Initially, cats with kidney stones displayed a less diverse intestinal microbiota. Antibiotic treatment reduced microbiota diversity in both groups. The absence of specific intestinal bacteria could lead to a loss of the functions these bacteria perform, such as oxalate degradation, which may contribute to the formation of calcium oxalate stones. This study confirms the presence of a distinct urobiome in cats with kidney stones, characterized by greater richness and diversity compared to healthy cats. These findings highlight the potential of microbiota modulation as a strategy to prevent renal lithiasis in cats.

## 1. Introduction

Urolithiasis in cats has significantly changed over the past two decades [1,2]. Starting in the 1990s, partly due to dietary modifications implemented to reduce the incidence of struvite-based lower urinary tract stones, the incidence of struvite lithiasis has decreased while the incidence of calcium oxalate lithiasis has increased [1,3,4,5,6,7]. As calcium oxalate stones form in the upper urinary tract [8], renal and ureteral lithiasis have become more prevalent while lower urinary tract lithiasis based on struvite has become less frequent. In cats, nephrolithiasis is more oxalocalcic than in humans; about 98% of feline kidney stones are calcium oxalate stones [7,9] compared to about 70–80% in humans [10,11].

Genetic factors alone cannot explain the sudden increase, within one or two generations, in the incidence of a disease. A rapid increase in the incidence of a disease can be observed in infectious diseases, underlying the relevance of examining whether microbial agents are involved in the pathogenesis of lithiasis. Recently, alongside genetic and environmental factors, a new player in health has been identified—the microbiota. The microbiota is defined as the community of microorganisms that exist in different parts of a living being. It is composed of bacteria, archaea, viruses, fungi, and protists. One of the mechanisms involved in the formation of calcium oxalate stones in kidneys is hyperoxaluria, leading to urine oversaturation with oxalate [12]. Oxalate is naturally present in the diet (it is a ubiquitous compound in plants), but mammals, including dogs, cats, and humans (except for the guinea pig) do not produce enzymes to metabolize it. Instead, they outsource this function to the bacteria of their intestinal microbiota [13]. *Oxalobacter formigenes* is the most studied bacterium for its ability to degrade oxalate [14,15,16,17,18,19,20], but its importance has been questioned [20]. Other bacteria such as Lactobacilli and *Bifidobacterium* use oxalate as an energy source, they are called oxalotrophic bacteria [20,21,22]. These bacteria degrade oxalate through the expression of two enzymes, Formyl-CoA transferase and Oxalyl-CoA decarboxylase [23,24,25,26]. An entire network of bacteria called “Oxalate Metabolizing Bacterial Species” (OMBS) is involved in oxalate metabolism [13,20,25,27,28,29,30]. By reducing the digestive absorption of oxalate, this bacterial network decreases the urinary excretion of oxalate, thereby lowering the risk of forming calcium oxalate stones. Conversely, in the absence of this network, a higher concentration of oxalate remains in the intestinal lumen, leading to increased intestinal absorption; if the concentration of oxalate in the blood exceeds metabolic capacities, an excessive amount of oxalate is excreted in the urine. In cases of hypersaturation, the oxalate can bind with urinary calcium, leading to the formation of calcium oxalate crystals in the urine. These crystals are likely to aggregate and form kidney stones. Given that bacteria may play a role in the pathogenesis of nephrolithiasis, it is relevant to investigate the consequences of antibiotic use on the microbiota.

The involvement of the intestinal and urinary microbiota in the formation of calcium oxalate stone has been studied in humans [22,31,32], in rats [33,34,35,36,37,38], and in dogs [27,39], but not in cats. We hypothesized the following: (1) that mechanisms involving the microbiota, similar to those suspected in humans, might contribute to the formation of kidney calcium oxalate stones in cats, and (2) the use of antibiotics could lead to dysbiosis and thereby increase the risk of calcium oxalate stone formation. The primary objective of this study was to compare the intestinal and urinary bacterial microbiota of a population of cats spontaneously presenting with kidney stones with that of a population of healthy cats. The second objective of this study was to examine the impact of antibiotic therapy on the composition of the intestinal and urinary bacterial microbiota in both populations.

## 2. Materials and Methods

### 2.1. Studied Population

#### 2.1.1. Recruitment of Cats

The Nutrition Service of our institution (Oniris, National Veterinary School of Nantes, France) maintained a cattery housing about one hundred domestic cats (*Felis catus*) designated for studying the qualities of certain foods or dietary supplements. Among these cats, all domestic shorthair cats that were sterilized before puberty and acquired healthy at the age of one year, some spontaneously developed kidney stones in the years following their arrival, on average at about the age of 7 years. Some of these cats with lithiasis died or were euthanized due to renal failure or severe pain; others, showing few or no symptoms, were retired and made available for adoption. For the present study, 9 asymptomatic neutered female cats with lithiasis were enrolled. Additionally, 9 control cats (7 neutered females and 2 castrated males) from the same colony, living under identical conditions and free from lithiasis, were enrolled to form a control group, making a total of 18 cats.

#### 2.1.2. Living Conditions

All cats were fed the same commercial senior dry food (Mature Consult Balance S/O, Royal Canin^®^, Aimargues, France) (See Supplementary Data for diet composition) for at least a year at a consistent daily ration of 50 g, served in one meal. They had unrestricted access to water and were maintained on a 12 h light/dark cycle. Throughout the year, the cats were housed in large aviaries comprising 8 individuals that could interact freely; the cats with lithiasis and the healthy cats were not separated.

#### 2.1.3. Health Status

The cats were up to date with their vaccination and deworming schedules. Apart from early sterilization, no animal had undergone any surgical procedures or any medication, including antibiotics, prior to the initiation of this study. Blood chemistry analyses were conducted to evaluate the stage of any potential kidney disease.

#### 2.1.4. Inclusion Criteria

The diagnosis of renal lithiasis or the absence of renal lithiasis was made using radiography and ultrasound. To be retained, cats with lithiasis had to present with one or more kidney stones visible on radiography in at least one kidney, and the presence of the stones had to be confirmed by ultrasound. Cats from the control group were considered healthy if no stone of the upper urinary tract was visible on radiography or ultrasound.

Given the studied species (domestic cat), the location of the stones (in the upper urinary tract), the radiological density of the stones, and their small size, the observed stones were considered to be calcium oxalate stones.

#### 2.1.5. Exclusion Criteria

The following were not included in this study:

- privately owned cats diagnosed with lithiasis,
- cats presumed healthy but whose renal architecture was not confirmed as normal on ultrasound,
- cats that had received antibiotic treatment prior to the study,
- cats with a significant pathological history,
- cats fed a diet different from that given to the study group,
- cats that had received prebiotics, probiotics, or postbiotics before the study.

The presence of ureteral or bladder stones was not an exclusion factor.

#### 2.1.6. Follow-Up

Although the study itself lasted only 28 days, the cats were monitored for a period of 600 days. If significant clinical signs appeared in cats with lithiasis (pain, loss of appetite, weight loss, behavioral changes), they were either offered for adoption after receiving care or euthanized in the absence of a reasonable therapeutic option.

### 2.2. Sample Collection

#### 2.2.1. Collection of Fecal Samples

The study adhered to the main recommendations of the first international consortium for microbiome in urinary stone disease–MICROCOSM–in human [40]. Fecal samples were collected immediately after spontaneous defecation to avoid alterations of the microbiome due to exposure to ambient air. Three samples were taken two weeks apart. On the day of each sampling (D0, D14, and D28), cats were isolated individually to ensure the collection of their specific fecal sample. Several cages were available in the isolation room; the front and sides were clear, allowing the cats to maintain visual contact with one another. Each cage had a surface area of 1.5 m^2^, enhanced by two resting shelves each measuring 0.19 m^2^. The cages, standing 140 cm high, were enriched with wall-mounted scratching posts and toys. No bedding was present in the isolation cage to limit contamination of the fecal sample by urine. The cats were observed to defecate in one corner of the cage and urinate in another, with urine being directed into an external container to minimize fecal sample contamination. On the day of collection, the cats were observed until they defecated. As soon as a cat had defecated, a fresh fecal sample weighing more than 5 g was collected with sterile forceps and immediately aliquoted into a sterile tube for analysis of the intestinal microbiota. The fecal samples were frozen immediately after collection and stored at −80 °C. Once all samples were collected, they were transported on dry ice in a single shipment to the laboratory for analysis. After thawing, the laboratory sampled the center of each specimen, thereby avoiding analysis of the stool periphery.

#### 2.2.2. Collection of Urine Samples

The urinary microbiota was studied using urine collected via ultrasound-guided cystocentesis to prevent contamination during urethral catheterization. Urine was collected on D0, D14, and D28 from all 18 cats. When necessary, cat sedation was carried out using a combination of medetomidine (10 μg/kg) and butorphanol (0.1 mg/kg) administered intramuscularly. A volume of 5 mL of urine was collected at each session and stored aseptically in a sterile tube. To preserve bacterial DNA, an equal volume of a DNA stabilizer (DNA/RNA Shield^TM^, Ozyme, Saint-Cyr-l’École, France) was added to the samples. The urine was kept at 4 °C and immediately transported to the laboratory for analysis. The urine samples were not frozen to prevent any potential alteration of the DNA within the sample due to freezing and thawing cycles.

#### 2.2.3. Collection of Calculi

If a cat suffering from lithiasis passed away, its stones were collected from the kidneys during autopsy, placed in plastic pots, then shipped to the Minnesota Urolith Center (USA) to analyze their composition. The quantitative mineral analysis was performed using optical crystallography and infrared spectroscopy.

### 2.3. Antibiotic Therapy

Following the initial samplings at D0, antibiotic therapy was initiated. The choice of antibiotic was made for practical reasons as follows: administering 36 oral tablets daily to occasionally unruly cats was challenging, and ensuring the complete dosage was uncertain. Therefore, the decision was made to use cefovecin (Convenia^®^, Zoetis, Malakoff, France), a third-generation cephalosporin marketed exclusively in veterinary medicine. Cefovecin has a pharmacological property that made it particularly interesting for this protocol—its extremely long half-life (5.5 days), ensuring that the plasma concentration of cefovecin remains high for 14 days following a single subcutaneous injection at the therapeutic dose. This feature has eliminated the uncertainties associated with oral intake and intestinal absorption. Moreover, cefovecin has a broad spectrum. In cats, it is indicated for the treatment of skin wounds and abscesses of the soft tissues associated with *Pasteurella multocida*, *Fusobacterium* spp., *Bacteroides* spp., *Prevotella oralis*, *Streptococci β-hemolytic*, and/or *Staphylococcus pseudintermedius*, and in the treatment of urinary tract infections associated with *Escherichia coli*.

**Figure 1:**
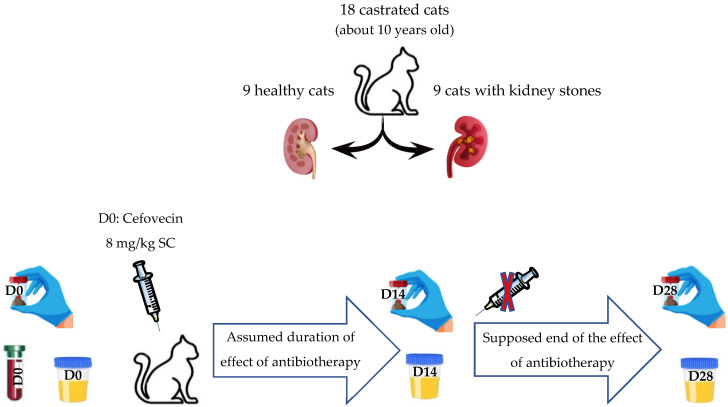
Timeline of the in vivo protocol of the study.

Cats were subcutaneously administered a single injection of 8 mg/kg of cefovecin (which is 0.1 mL/kg of Convenia^®^) on D0, following the collection of the initial fecal and urine samples. A second sampling of feces and urine was performed on the 14th day (D14) to observe the impact of antibiotic therapy on the intestinal and urinary microbiota. A third and final sampling was conducted on the 28th day (D28), which is 14 days after the expected end of the antibiotic effect, to evaluate the resilience of the intestinal and urinary microbiota after discontinuing the antibiotic therapy (Figure 1).

### 2.4. DNA Isolation and 16S rRNA Amplicon Sequencing

#### 2.4.1. High-Throughput Bacterial DNA Sequencing Techniques Used

The bacteria in the intestinal microbiota are largely uncultivable, which is why the microbiota is studied using metagenomics, a DNA sequencing technique [41]. In this study, the 16S rRNA pyrosequencing was used to identify the bacterial composition of the microbiota, without resorting to global metagenomics. The 16S rRNA is present only in bacteria; hence, only the bacterial microbiota was addressed, while archaea, viruses, fungi and protists were not studied.

#### 2.4.2. DNA Extraction

DNA was extracted using the ZymoBIOMICS™ 96 MagBead DNA Kit (Zymo Research Corp., Tustin, CA, USA) following a protocol with dual cell lysis (mechanical and chemical). DNA isolation was carried out on a KingFisher Flex automated station (Thermo Fisher Scientific Inc., Boston, MA, USA) according to the manufacturer’s instructions. DNA was quantified by fluorimetry using a Qubit^®^ 2.0 (Thermo Fisher Scientific, USA).

#### 2.4.3. 16S rRNA Gene Amplification and Sequencing [42]

The V3–V4 region of the gene encoding the bacterial 16S ribosomal RNA was amplified by polymerase chain reaction (PCR) using primers 341F and 785R. The amplicons were cleaned up using magnetic AMPure XP beads (Beckman Coulter, Villepinte, France) before adding dual indices and sequencing adapters using the Illumina Nextera XT Index kit (Illumina, San Diego, CA, USA). Each library was cleaned up and quantified by fluorimetry (Qubit^®^ 2.0 Fluorometer), normalized, and pooled. The pooled library was denatured before sequencing (2 × 250 paired-end, v2 chemistry) using an Illumina MiSeq Sequencing system (Illumina, San Diego, CA, USA).

#### 2.4.4. Data Processing

The sequences were analyzed using a bioinformatic pipeline developed by Biofortis based on Dadaist2 software. Basically, after demultiplexing the indexed reads, single read sequences were paired for each sample into longer fragments and cleaned. After quality-filtering and sequencing error modeling, amplicon sequence variants (ASV) were obtained. A taxonomic assignment of these ASV was performed in order to determine bacterial community profiles.

#### 2.4.5. Statistical Analyzes

Linear Discriminant Analysis (LDA) score-based graphs were generated via the Huttenhower Galaxy web application via Linear Discriminant Analysis Effect Size (LEfSe) module. A Principal Component Analysis (PCA) plot was generated and OTU-based diversity indices were calculated with the software PAST (PAleontological Statistics) 4.10. A *p*-value below 0.05 was considered statistically significant.

To determine whether mortality in cats with lithiasis was significantly different compared to healthy cats, a binomial exact test was conducted using a reference probability of 1. The calculation was carried out with R software, version 4.2.2, using the “binom.test” function [43].

## 3. Results

### 3.1. Studied Population

This study enrolled nine cats with spontaneous renal lithiasis presumed to be calcium oxalate and nine healthy control cats. The group of cats with lithiasis included nine females and the healthy cat group included seven females and two males; all cats were sterilized before puberty. The ages at D0 ranged from 9.7 to 11.5 years (3541 days–4213 days). The median age at D0 was 3572 days (±259 days), which is about 10 years. The median weight of the cats was 4.075 kg (±889 g).

**Figure 2:**
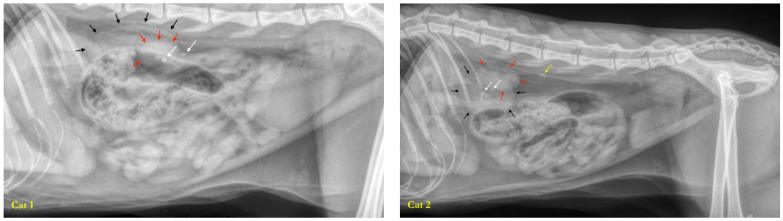

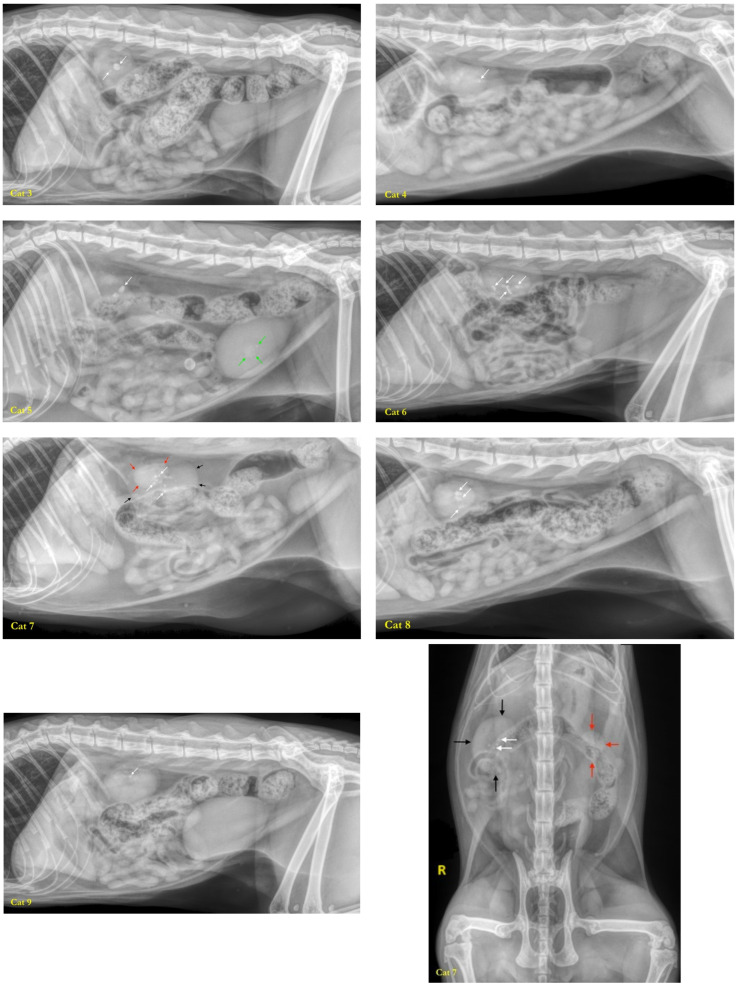
Abdominal radiographs of cats with one or more kidney stones (white arrows) (profile view of cats 1 to 9 in reading direction and front view of cat 7). Cat 2 had a ureteral stone (yellow arrow). Cat 5 had several small stones in the bladder (green arrows). Renal asymmetry can be observed in certain profile views (cats 1, 2, and 7), with the contours of the larger kidney indicated by black arrows, and the contours of the smaller kidney indicated by red arrows.

As an inclusion criterion, all cats had at least one stone in one of the two kidneys (Figure 2). One cat (cat 2) had a stone in the ureter on the same side as the kidney with stones. One cat (cat 5) had bladder stones. Six cats exhibited unilateral renal lithiasis (cats 1, 2, 6, 7, 8, and 9), and the remaining three cats had bilateral renal lithiasis (cats 3, 4, and 5). Only one cat (cat 9) had a single renal stone; all others had multiple renal stones. Renal asymmetry with atrophy of one kidney was observed in five cats (cats 1, 2, 3, 5, and 7), three cats exhibited no asymmetry and had kidneys of normal size (cats 4, 5, and 9) and one cat had atrophy of both kidneys (cat 8). Small kidneys are considered to be kidneys that have involuted following an occult episode of ureteral obstruction.

All the cats in the study, except for one, had azotemia within normal ranges at D0. One cat (cat 8) had azotemia slightly above the normal values (uremia at 1 g/L (range: 0.2–0.4) and creatininemia at 22 mg/dL (range: <16)); this cat died from renal failure at D165 and it was the only cat that had atrophy of both kidneys at the beginning of the study. Another cat from the lithiasis group died of renal failure in the tenth month of the study (cat 3, died at D318), he had bilateral lithiasis. A third cat was found dead one morning at D500, 18 months after the start of the study (cat 1), he also had bilateral lithiasis. In total, three cats among the nine with lithiasis died within the 500 days following the beginning of the study. None of the cats in the control group (healthy cats) had died at D600 (with D0 being 9 June 2022). The *p*-value obtained was <0.0001 (*p* = 2.2 × 10^−16^), indicating that mortality in the group of cats with lithiasis was significantly higher than in the group of healthy cats.

For the three cats with lithiasis that died, the body of the stones was composed of 100% calcium oxalate monohydrate.

All 18 cats provided fecal and urine samples that were deemed quantitatively sufficient for metagenomic analysis at D0, D14 and D28. The median pH of urine at D0 for healthy cats was 6.31 (min: 5.91; max: 6.75); the median pH of urine at D0 for cats with lithiasis was 6.28 (min = 6.02; max = 7.66).

No cat presented antibiotic-associated diarrhea.

### 3.2. Characterization of the Fecal Microbiota

#### 3.2.1. Alpha Diversity

**Figure 3:**
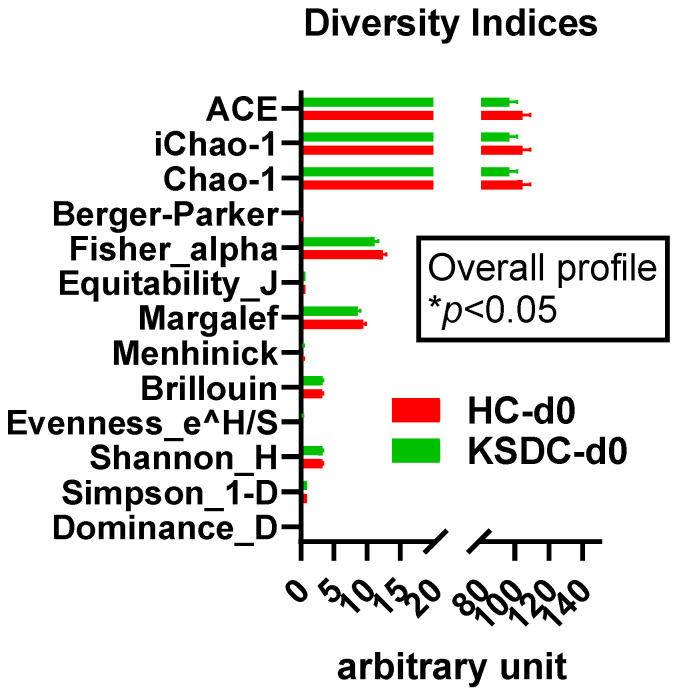
Comparison of the diversity indices between the healthy cats (HC) (in red) and the kidney stone diseased cats (KSDC) (in green) at day 0 (d0). * *p*-value < 0.05, 2-ANOVA followed by a two-stage linear step-up procedure of Benjamini, Krieger, and Yekutieli to correct for multiple comparisons by controlling the False Discovery Rate (<0.05).

At D0, alpha diversity was notably greater in the healthy cat group than in the cats with lithiasis. This was evidenced by the higher diversity indices in the healthy group when compared to those with lithiasis (Figure 3).

#### 3.2.2. Beta Diversity

##### Taxonomic Analysis of the Intestinal Microbiota Composition at D0 in the Two Cat Populations

**Figure 4:**
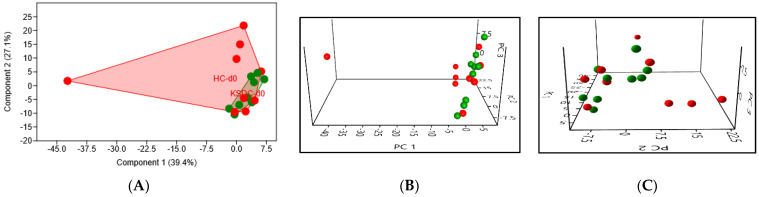
(**A**) 2D PCA graphic comparing, at D0, the intestinal microbiota of the two cat populations, healthy cats (HC) in red and kidney stone diseased cats (KSDC) in green. (**B**,**C**) Exploring the same previously described patterns with 3D PCA plotting.

PCA at D0 showed some differences in the intestinal microbiota composition between the two cat populations (Figure 4). In particular, healthy cats displayed a higher intragroup variance than those with lithiasis, whose group appeared more homogenous. 

##### Biodiversity of the Fecal Microbiota in the Two Cat Populations

**Figure 5:**
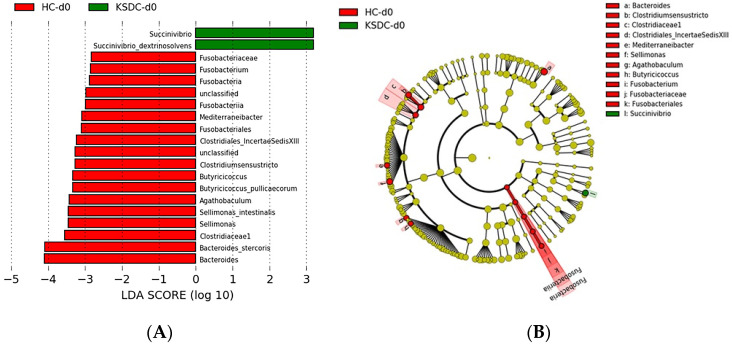
(**A**) Histogram of the LDA scores for differentially abundant bacterial clades in the fecal samples between healthy cats (HC) and kidney stone diseased cats (KSDC). Negative (red bars) LDA scores represent bacterial groups that are over-abundant in HC samples, while positive (green bars) represent bacterial groups that are overrepresented in the KSDC samples at day 0 (d0). LDA score also indicates the effective size and ranking of each differentially abundant taxon (LDA score > 3.0; *p*-value < 0.05). (**B**) Cladogram of phylogenetic relationships of bacterial lineages associated with healthy cats (HC) and kidney stone diseased cats (KSDC) at day 0 (d0): the taxonomic levels are represented by rings with phyla at the innermost and genera at the outermost ring, and each circle is a bacterial member within that level.

Comparing at D0, the intestinal microbiota of the healthy cats with that of the cats presenting with lithiasis, 16 and 2 taxa, respectively, were identified with LDA scores greater than three and a *p*-value < 0.05 (Figure 5).

LEfSe analysis at D0 showed that bacterial species *Bacteroides stercoris*, *Butyricicoccus pullicaecorum*, and *Sellimonas intestinalis* were more abundant in healthy cats than in cats with lithiasis. Similarly, bacteria belonging to the genera *Agathobaculum*, *Clostridium sensu stricto*, *Fusobacterium*, and *Mediterraneibacter* predominated in healthy cats. LEfSe analysis also showed a significantly higher abundance of bacteria belonging to the families *Clostridiaceae1*, *Clostridiales_IncertaeSedisXIII*, and *Fusobacteriaceae* in healthy cats compared to cats with lithiasis. Finally, bacteria belonging to the taxa Fusobacteriia and Fusobacteria were overrepresented in the healthy cats compared to cats with lithiasis. LEfSe analysis at D0 showed that the bacterial species *Succinivibrio dextrinosolvens* was significantly more abundant in cats with lithiasis than healthy cats.

### 3.3. Taxonomic Analysis of the Intestinal Microbiota Composition at D0, D14, and D28 in the Healthy Cats Population

To assess the impact of antibiotic therapy on the intestinal microbiota of healthy cats 14 days after its initiation and the ability of the intestinal microbiota to recover its initial composition 14 days after the supposed cessation of the antibiotic effect, i.e., 28 days after the start of antibiotic therapy, the composition of the intestinal microbiota at D0 (before the initiation of antibiotic therapy), at D14 (after 14 days of antibiotic therapy), and at D28 (14 days after the supposed end of cefovecin efficacy) was compared in the three sets of samples.

#### 3.3.1. Alpha Diversity

**Figure 6:**
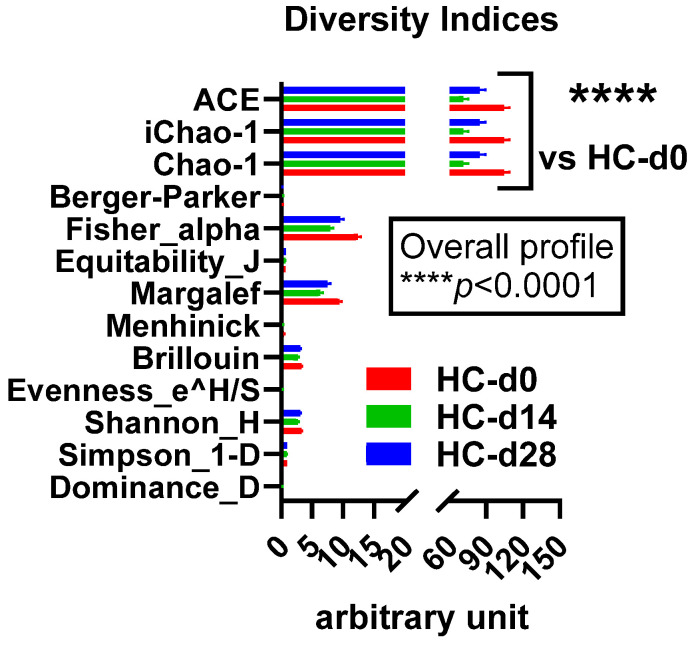
Comparison of the diversity indices among the healthy cats (HC) at day 0 (d0) (in red), day 14 (d14) (in green), and day 28 (d28) (in blue). **** *p* < 0.0001, 2-ANOVA followed by a two-stage linear step-up procedure of Benjamini, Krieger, and Yekutieli to correct for multiple comparisons by controlling the False Discovery Rate (<0.05).

In the healthy cats, the diversity indices were in general the highest at D0 and lowest at D14, with those at D28 showing intermediate values (Figure 6). These results indicate that bacterial richness and diversity were most significantly impacted 14 days after the initiation of antibiotic therapy (D14) and that these richness and diversity were not fully restored 14 days after the supposed cessation of the antibiotic efficacy (D28).

#### 3.3.2. Beta Diversity

**Figure 7:**
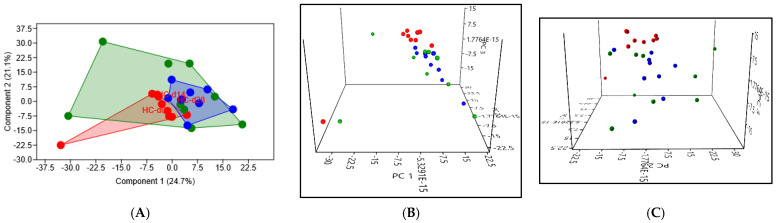
(**A**) 2D PCA graphic comparing the intestinal microbiota of the healthy cats at D0 (before the initiation of antibiotic therapy) in red, at D14 (after 14 days of antibiotic therapy) in green, and at D28 (14 days after the supposed cessation of the antibiotic efficacy based on cefovecin) in blue. (**B**,**C**) Exploring the same previously described patterns with 3D PCA plotting.

In the healthy cats, PCA revealed significant differences in the composition of the intestinal microbiota, with a *p*-value of 0.014 when comparing D0 to D14, and a *p*-value of 0.0009 when comparing D0 to D28 (Figure 7). The high intragroup variance at D14 suggests that the cats responded very variably to the antibiotic therapy. The low intragroup variance at D28 indicates a higher uniformity of response to the antibiotic therapy. The proximity of the points at D0 and D28 suggests relatively good resilience of the intestinal microbiota in healthy cats. 

**Figure 8:**
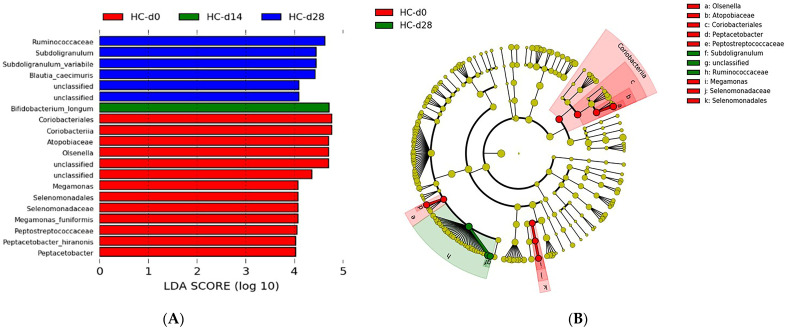
(**A**) Histogram of LDA scores for differentially abundant bacterial clades in the fecal samples between day 0 (d0), day 14 (d14), and day 28 (d28) in healthy cats (HC). Blue LDA scores represent bacterial groups over-abundant in the HC samples on day 28 after the beginning of antibiotic administration. Green bars represent bacterial groups overrepresented at day 14 after the beginning of the antibiotic therapy. Red bars represent bacterial groups over-abundant in HC fecal samples at day 0. LDA score also indicates the effective size and ranking of each differentially abundant taxon (LDA score > 4.0; *p*-value < 0.01). (**B**) Cladogram of phylogenetic relationships of bacterial lineages associated with healthy cats (HC) at day 0 (d0) and day 28 (d28). Note that HC-d14 samples have been analyzed in B as well, but they cannot appear since the outmost level shown by the cladogram is genus, whereas HC-d14 is characterized by a significantly higher abundance of *Bifidobacterium longum*, which is a species.

When comparing the composition of the intestinal microbiota at D0, D14, and D28 in the healthy cats, 13, 1, and 4 taxa were identified, respectively, with LDA scores greater than four and a *p*-value < 0.01 (Figure 8).

At D0, LEfSe analysis showed that bacteria belonging to the species *Peptacetobacter hiranonis* and *Megamonas funiformis* were more abundant compared to D14 and D28. Similarly, there was a significantly higher abundance of bacteria belonging to the genera *Olsenella* and the families *Atopobiaceae*, *Selenomonadaceae*, and *Peptostreptococcaceae*, as well as bacteria from the Coriobacteriales, Coriobacteriia, and Selenomonadales orders. At D14, the bacterial species *Bifidobacterium longum* was overrepresented in this group of healthy cats.

At D28, the bacterial species *Subdoligranulum variabile* and *Blautia caecimuris*, as well as the family *Ruminococcaceae*, were significantly more abundant.

### 3.4. Taxonomic Analysis of the Intestinal Microbiota Composition at D0, D14, and D28 in the Population of Cats with Lithiasis

#### 3.4.1. Alpha Diversity

**Figure 9:**
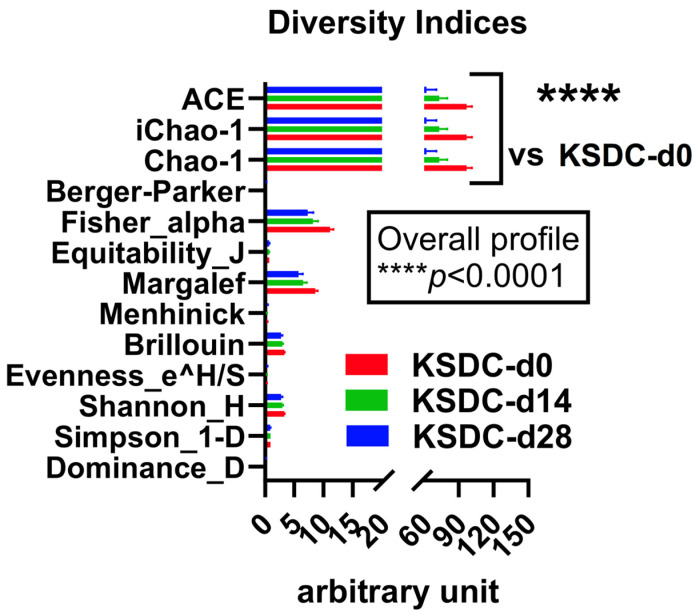
Comparison of the diversity indices among the kidney stone diseased cats (KSDC) at day 0 (d0) (in red), day 14 (d14) (in green), and day 28 (d28) (in blue). **** *p* < 0.0001, 2-ANOVA followed by a two-stage linear step-up procedure of Benjamini, Krieger, and Yekutieli to correct for multiple comparisons by controlling the False Discovery Rate (<0.05).

In cats with lithiasis, the diversity indices were highest at D0, decreased at D14, and were even lower at D28 (Figure 9). Therefore, unlike the healthy cats whose alpha diversity increased at D28 compared to D14, indicating some resilience of the microbiota, the cats with lithiasis, which initially had a poorer microbiota than that of healthy cats, experienced a more lasting and stronger impact from antibiotic therapy; there was no resilience at D28, and on the contrary, alpha diversity decreased further.

#### 3.4.2. Beta Diversity

**Figure 10:**
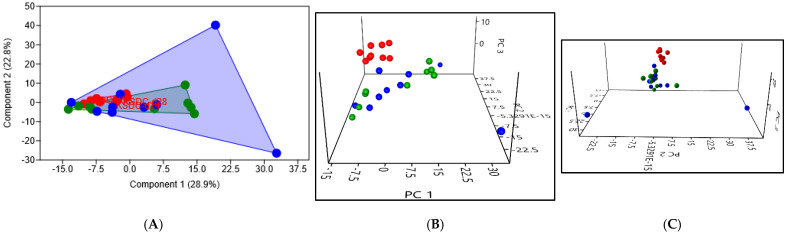
(**A**) 2D PCA comparing the intestinal microbiota of cats with lithiasis at D0 (before the initiation of antibiotic therapy) in red, at D14 (after 14 days of antibiotic therapy) in green, and at D28 (14 days after the supposed cessation of the efficacy of antibiotic therapy based on cefovecin) in blue (**B**,**C**). Exploring the same previously described patterns with 3D PCA plotting.

In cats with lithiasis, PCA revealed even more significant differences in the composition of the intestinal microbiota, with a *p*-value of 0.0042 when comparing D0 to D14, and still a *p*-value of 0.0009 when comparing D0 to D28 (Figure 10). The high intragroup variance observed at D28 reveals a still very different composition of the intestinal microbiota for some cats, 14 days after the supposed cessation of the antibiotics efficacy.

**Figure 11:**
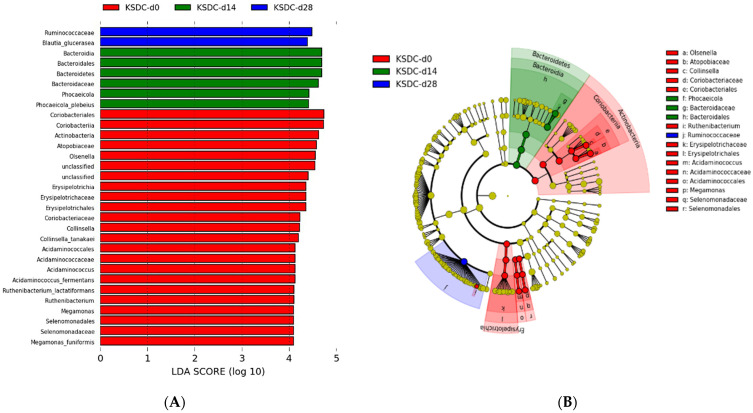
(**A**) Histogram of LDA scores for differentially abundant bacterial clades in fecal samples between day 0 (d0), day 14 (d14), and day 28 (d28) in cats with kidney stones (KSDC). Blue LDA scores represent bacterial groups that are overabundant in KSDC samples on day 28 after the start of antibiotic administration. Green bars represent bacterial groups overrepresented at day 14 after the start of the antibiotic therapy. Red bars represent bacterial groups that are overabundant in KSDC fecal samples at day 0. LDA score also indicates the effective size and ranking of each differentially abundant taxon (LDA score > 4.0; *p*-value < 0.05). (**B**) Cladogram showing the phylogenetic relationships of bacterial lineages associated with cats with kidney stones (KSDC) at day 0 (d0), day 14 (d14), and day 28 (d28).

In comparing the intestinal microbiota composition at D0, D14, and D28 in cats with lithiasis, 21, 6, and 2 taxa were identified, respectively, with LDA scores greater than 4 and a *p*-value < 0.05 (Figure 11). At D0 compared to D14 and D28, LEfSe analysis showed a higher abundance for the following bacterial species: (i) bacteria belonging to the species *Collinsella tanakaei*, *Acidaminococcus fermentans*, *Ruthenibacterium lactatiformans*, and *Megamonas funiformis*; (ii) bacteria belonging to the genera *Olsenella* and the families *Atopobiaceae*, *Erysipelotrichaceae*, *Coriobacteriaceae*, *Acidaminococcaceae*, and *Selenomonadaceae*; and (iii) bacteria from orders Actinobacteria, Coriobacteriales, Coriobacteriia, Erysipelotrichales, Erysipelotrichia, Acidaminococcales, and Selenomonadales.

At D14, the bacterial species *Phocaeicola plebeius* (formerly known as *Bacteroides plebeius*) and bacteria belonging to the taxa *Bacteroidaceae*, Bacteroidales, Bacteroidia, and Bacteroidetes were overrepresented compared to D0 and D28. 

At D28, the bacterial species *Blautia glucerasea* and the family *Ruminococcaceae* were overrepresented compared to D0 and D14.

### 3.5. Characterization of the Urinary Microbiota

#### 3.5.1. Available Results

The 18 cats provided a urine volume of 5 mL deemed sufficient for metagenomic analysis; however, results were obtained at Day 0 only for 10 cats, 5 healthy cats, and 5 cats with lithiasis. Due to insufficient bacterial loads, the Day 0 analysis failed in eight cats, four of which belonged to the healthy cat group and four to the group with lithiasis; the quantity of the sequences obtained after sequencing was equal to that obtained in the negative controls, thus indistinguishable from sequencing noise. On Days 14 and 28, after the use of antibiotics, none of the urine samples returned a microbiota.

#### 3.5.2. Alpha Diversity

**Figure 12:**
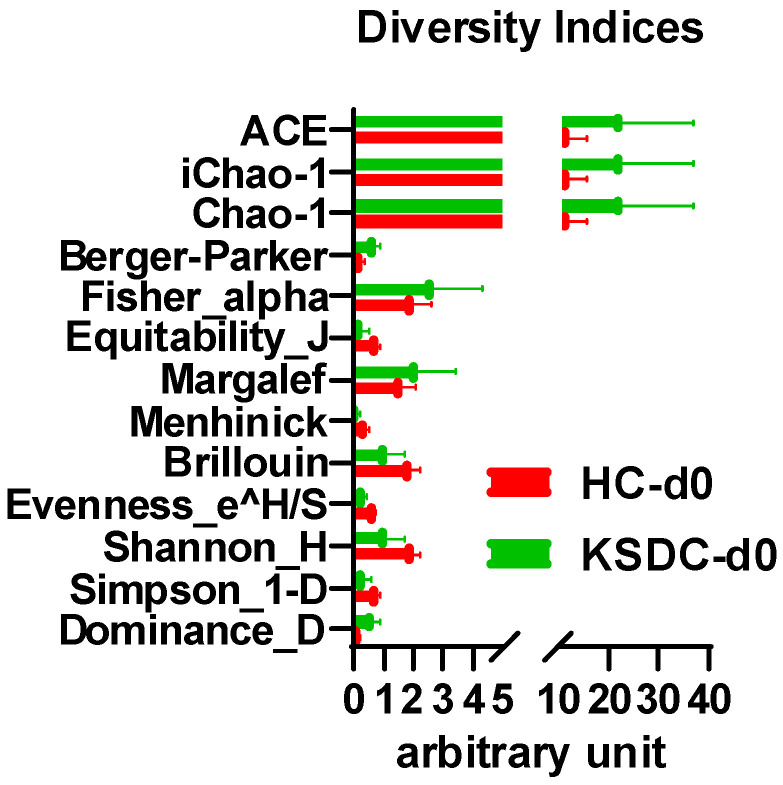
Comparison of the diversity indices between the healthy cats (HC) and the kidney stone diseased cats (KSDC) at D0.

The diversity indices ACE, iChao-1, Chao-1, Berger–Parker, Fisher–alpha, Margalef, and Dominance_D were higher, though not significant, for the group of cats with lithiasis compared to the healthy cat group; by contrast, the diversity indices were slightly lower for Equitability_J, Menhinick, Brillouin, Evenness_e^H/S, Shannon-H, and Simpson_1-D. (Figure 12). Thus, there appears to be a richer and more varied bacterial population in the urine of cats with lithiasis. 

#### 3.5.3. Beta Diversity

**Figure 13:**
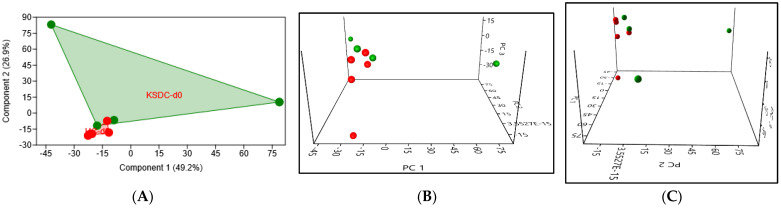
(**A**) 2D PCA graphic comparing the urinary microbiota composition of a healthy cat population (in red) and a population of cats with lithiasis (in green), *p*-value = 0.03. (**B**,**C**) Exploring the same previously described patterns with 3D PCA plotting.

PCA in the urine at Day 0 revealed a significant difference in the urinary microbiota composition between the two cat populations (*p* = 0.03), mainly due to cats with lithiasis showing a great intragroup variance (Figure 13). 

**Figure 14:**
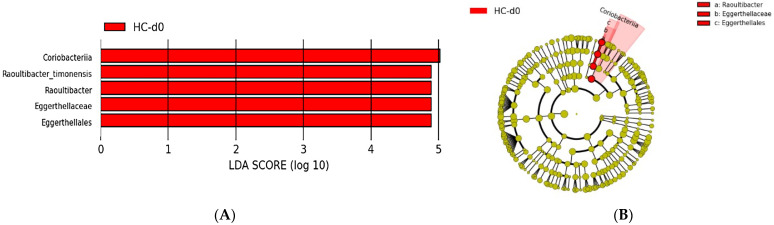
(**A**) Histogram of LDA scores for differentially abundant bacterial clades in the urinary samples between healthy cats (HC) and kidney stone diseased cats (KSDC). Positive (red bars) LDA scores represent bacterial groups over-abundant in HC samples. LDA score also indicates the effective size and ranking of each differentially abundant taxon (LDA score > 4.0; *p*-value < 0.01). (**B**) Cladogram of the phylogenetic relationships of bacterial lineages associated with healthy cats (HC) and kidney stone diseased cats (KSDC) (*p*-value < 0.01). Note that KSDC-d0 samples have been analyzed in A and B as well, but they do not appear since diversity indices are likely not reflecting these taxa that are found higher here. Also, the bacteria *Raoultibacter timonensis* shown in (**A**), which is a species, does not appear in (**B**) since the outmost level shown by the cladogram is the genus.

Comparing the urinary microbiota composition between the healthy cats and cats with lithiasis, we identified five bacterial taxa overrepresented in the healthy cats, with LDA scores greater than four and a *p*-value < 0.01 (Figure 14).

TheLEfSe analysis showed that the bacterial species *Raoultibacter timonensis* was significantly more abundant in the healthy cat group compared to the group of cats with lithiasis and that there was an overrepresentation of bacteria belonging to the class Coriobacteriia, more specifically to the order Eggerthellales and the family *Eggerthellaceae*. Therefore, these bacteria could have a protective effect against the risk of kidney stone formation. 

## 4. Discussion

This study is the first to compare the intestinal and urinary microbiota of cats with kidney stones, presumed to be calcium oxalate calculi, to the intestinal and urinary microbiota of healthy control cats; it also examines the impact of antibiotic therapy on the microbiota in these two populations.

### 4.1. Study of the Intestinal Microbiota in Patients with Kidney Stones

#### 4.1.1. Previous Studies

In veterinary medicine, two complementary studies undertaken by the same authors compared the intestinal microbiota of dogs with calcium oxalate stones to that of healthy dogs [17,27]. The location of the stones was not being specified but the calculi were surgically extracted, so it is very likely that they were bladder stones. Regarding calcium oxalate stones, it is likely that they originated in the kidneys, so the underlying mechanisms might be similar to those observed in cats.

In human medicine, eight studies have compared the composition of the intestinal microbiota of patients with renal lithiasis to that of healthy individuals [19,20,44,45,46,47,48,49], they account for 356 stone patients and 347 control [32]. All these studies used 16S rRNA sequencing and one study also used the shotgun sequencing [20]. A pilot study was conducted among children but there was no comparison with a control group [50].

#### 4.1.2. Alpha Diversity

In this study, alpha diversity was more pronounced in the group of healthy cats than in the group of cats with lithiasis. This is consistent with observations made for other diseases; an abundant and varied microbiota is more often associated with good health whereas a microbiota with limited diversity is often linked to poor health outcomes. The absence of certain bacteria is frequently correlated with the loss of specific functions these bacteria performed, which in turn is associated with diseases.

In a veterinary study, conducted not in cats but in dogs, comparing the intestinal microbiome of five dogs with oxalocalcic lithiasis to that of five healthy dogs [27], alpha diversity was not different in the two groups.

In humans, contradictory results have been obtained regarding alpha diversity [32]. Only one study noted a significant reduction in alpha diversity (Chao1 index) in kidney stone patients when compared to a control group (*p* = 0.02) [20]. Conversely, three studies found no difference between the kidney stones group and the control group [44,45,46], and one study reported an increase in alpha diversity in kidney stone patients, with higher value observed for the Simpson, Ace, and Chao indices [49].

The observations from our study suggest a link between low intestinal microbial richness and diversity and the risk of kidney stone formation in cats, that cats with lithiasis might lack oxalotrophic bacteria which would lead to poorer degradation of oxalate in the gut, greater intestinal absorption of oxalate, and subsequently hyperoxaluria. Given the lack of consistent findings in humans and dogs, further studies are necessary to confirm this observation.

#### 4.1.3. Beta Diversity

In this study, PCA at Day 0 revealed a difference in the composition of the intestinal microbiota between the healthy cats and those with lithiasis. In humans, five out of eight studies examined beta diversity, and four of them [20,44,46,49] also reported differences in the composition of the intestinal microbiota between patients with lithiasis and controls.

In healthy cats, at Day 0, the bacterial species *Bacteroides stercoris*, *Butyricicoccus pullicaecorum* and *Sellimonas intestinalis*, the genera Agathobaculum, *Clostridium sensu stricto*, *Fusobacterium* and *Mediterraneibacter*, the families *Clostridiaceae1*, *Clostridiales_IncertaeSedisXIII* and *Fusobacteriaceae* and the taxa Fusobacteriia and Fusobacteria were more abundant compared to cats with renal lithiasis. This suggests that these bacteria may play a protective role against the formation of calcium oxalate stones, and conversely, their absence could be a risk factor for kidney stone formation.

In cats with lithiasis, on Day 0, there was an overrepresentation of the species *Succinivibrio dextrinosolvens*, an anaerobic, Gram-negative bacterium belonging to the Pseudomonadata phylum (formerly called Proteobacteria). This observation suggests that this bacterial species could be associated with a risk of renal lithiasis in cats, but the role of *Succinivibrio dextrinosolvens* in urinary lithiasis had not been mentioned in previous studies and therefore remains to be elucidated. 

In veterinary medicine, a study conducted in dogs showed that the relative abundance of Firmicutes was higher in dogs with calcium oxalate stones than in healthy dogs [27]. Studies in humans have shown great heterogeneity in species distribution [32]. A prevailing trend, observed in four out of eight published studies, was that *Bacteroides* were more abundant in kidney stone patients compared to control [19,20,46,47]. 

The results of this study do not show a higher detection of *Oxalobacter formigenes* in healthy cats compared to those with lithiasis. This observation is consistent with observations made in humans [20,44,45,47], confirming the need to move beyond the reductive view that lithiasis development is solely linked to the absence or rarity of *Oxalobacter formigenes*; rather, an entire bacterial community is involved in the intestinal degradation of oxalate.

### 4.2. Impact of Antibiotic Therapy on the Intestinal Microbiota

#### 4.2.1. Choice of Cefovecin

The choice of cefovecin was made for practical reasons but this particular antibiotic is probably less involved in the disturbances of the feline population microbiota as its prescription is restricted by law in our country ever since cefovecin was listed among critical antibiotics. Its use is prohibited unless there is evidence of a bacterium sensitive to this antibiotic. However, it is very likely that observations made with cefovecin could be extrapolated to other antibiotics.

#### 4.2.2. Alpha Diversity

Significant changes in the composition of the intestinal microbiota were observed after the administration of cefovecin. A reduction in the richness and diversity of the intestinal and urinary microbiota in both populations of cats—healthy cats and cats with kidney stones—was observed after the implementation of antibiotic therapy. Several studies have shown that antibiotic treatment reduces microbial diversity in the digestive tract by a quarter to a third [51,52].

#### 4.2.3. Beta Diversity

On Day 14, in healthy cats, an overrepresentation of the bacterium *Bifidobacterium longum* was observed. *Bifidobacterium longum* does not participate in the degradation of oxalate since the genes encoding the two enzymes, Formyl-CoA transferase and Oxalyl-CoA decarboxylase, were not found in the genome of this bacterium [53]. In a study examining 12 strains of Bifidobacteria [54], 3 strains were found incapable of degrading oxalate, one of which was *Bifidobacterium longum*. The overrepresentation of this bacterial species on Day 14 in healthy cats signifies colonization by bacteria that are not oxalotrophic following administration of cefovecin.

On Day 14, in cats with lithiasis, the bacterial species *Phocaeicola plebeius* and bacteria belonging to the taxa *Bacteroidaceae*, Bacteroidales, Bacteroidia, and Bacteroidetes were overrepresented in the group of cats with lithiasis compared to healthy cats. The overrepresentation of *Bacteroides* (*Phocaeicola plebeius* belongs to the phylum Bacteroidetes) aligns with observations made in four studies conducted in humans [19,20,46,47]; *Bacteroides* is therefore a bacterial genus that can be considered a biomarker for calcium oxalate kidney stones. The use of antibiotics in healthy cats has made the composition of their microbiota similar to that observed in human patients with lithiasis.

The results obtained on Day 14, for both healthy cats and those with renal lithiasis, demonstrated that antibiotic therapy had disrupted the composition of the intestinal microbiota at the expense of the Firmicutes phylum, in favor of the Actinobacteria phylum for healthy cats (with *Bifidobacterium longum*) and in favor of the Bacteroidetes phylum for cats with lithiasis. This disruption led to an increase in the Bacteroidetes/Firmicutes ratio in cats with lithiasis mainly due to the proliferation of Bacteroidetes compared to Firmicutes; this increase in the Bacteroidetes/Firmicutes ratio is a short-term effect of antibiotics on the human intestinal microbiota [52]. Dozens of studies have investigated the effects of antibiotics on the composition of the intestinal microbiota [55,56].

On Day 28, a resemblance of overrepresented bacterial taxa in both groups was observed. In healthy cats, bacterial species *Subdoligranulum variabile* and *Blautia caecimuris*, as well as the *Ruminococcaceae* family, all parts of the Firmicutes phylum, were overrepresented. In cats with lithiasis, the bacterial species *Blautia glucerasea* and the *Ruminococcaceae* family, also parts of the Firmicutes phylum, were overrepresented. Thus, 28 days after the start of antibiotic therapy, the composition of the intestinal microbiota of healthy cats resembled that of cats with lithiasis on Day 28. In both groups, the Firmicutes phylum was predominant and an overrepresentation of the *Ruminococcaceae* family and the genus *Blautia* was observed.

The bacterial genus *Subdoligranulum* is considered a risk factor for the development of calcium oxalate lithiasis [57]. The overrepresentation of this genus on Day 28 in healthy cats supports our initial hypothesis that antibiotic therapy contributes to the recent increase in the incidence of feline lithiasis, partly explained by the medicalization of cats living in industrialized countries.

### 4.3. Resilience

The intestinal microbiota did not return to its original composition even by Day 28, which is 14 days after the presumed end of the antibiotic therapy effect. One explanation is that the effect of cefovecin does not wear off in 14 days, but persists well beyond; in one study, during the period of 21 days following the administration of cefovecin, only half of the administered radiolabeled dose was excreted through urine and around a quarter through feces [58]. The analyses of the microbiota on Day 28 are therefore probably not an accurate reflection of the intestinal microbiota resilience.

In the case of incomplete resilience, it is logical to expect a reduction or even disappearance of certain functions performed by the missing bacteria, possibly the oxalotrophic bacteria, which could promote the development of calcium oxalate stones [59,60,61]. Studies in humans showed that oral antibiotic use was associated with an increased risk of kidney stones [62,63,64].

### 4.4. Study of the Urinary Microbiota

The present study is the first to examine the composition of the urinary microbiota in cats with kidney stones.

#### 4.4.1. Urine Is Not Sterile in Cats

Bacterial loads were found to be low in the urine such that the analysis did not yield a microbiome in 8 out of 18 cats. In human medicine as well, it is sometimes observed that because of the low microbial biomass present in some samples, successful 16S rRNA gene amplification was not achieved in all collected samples [42]. However, in the 10 cats showing no signs of urinary infection, bacterial populations were identified in the urine of 5 healthy cats and 5 cats with lithiasis.

In humans, contrary to a long-standing belief, it is now accepted that urine is not sterile; it harbors a microbiota—termed the urobiome—even though this microbiota is much less abundant than in the gut [65,66,67,68,69]. This paradigm shift must now be extended to cats, previously considered to have particularly sterile urine. Two earlier studies had not detected a urobiome in cats; the first involved 10 healthy cats [70] and the second examined 38 culture-negative urine samples from cats with feline lower urinary tract disease alongside 43 culture-negative samples from healthy control cats [71]; for the second study, the authors admitted potential methodological limitation [71]. The existence of a urobiome was demonstrated in a study involving healthy cats, cats with chronic renal failure, and cats with idiopathic cystitis [72]. To our knowledge, the present study is the second that confirms the existence of a urobiome in cats. The existence of a urobiome was also demonstrated in dogs [73,74,75].

#### 4.4.2. A Different Urobiome in the Stone Formers Cats

The main indices of diversity, including notably Chao-1, revealed a richer and more varied urobiome in cats with lithiasis compared to healthy cats. PCA showed a difference in the composition of the urinary microbiota between the two populations of cats (*p*-value = 0.03). Thus, bacteria from the urinary microbiota could be involved in the pathogenesis of oxalocalcic lithiasis. 

In our study, the LEfSe analysis showed an overrepresentation of the bacterial species *Raoultibacter timonensis* and bacteria belonging to the class Coriobacteriia in healthy cats; by contrast, different observations have been made in humans [64,68,76].

Antibiotic therapy had a significant impact on the urinary microbiome because at D14 and D28, that is after the use of an antibiotic, none of the samples yielded a urobiome.

In the only available study in dogs, comparing the urinary microbiota of miniature schnauzers with calcium oxalate bladder stones (*n* = 9) to that of healthy miniature schnauzers (*n* = 10) [39], alpha and beta diversities were not different in the two groups. It is conceivable that in this canine model where racial predisposition is strong, the genetic risk factor may be predominant, whereas in the feline model we studied, the microbiota plays a more significant role.

In humans, several studies have shown a difference in the composition of the urobiome in patients with kidney stones compared to individuals without it [64,68,76,77]. It is suspected that the urinary microbiota may play an even more significant role than the intestinal microbiota in the formation of kidney stones; the same could be true for cats. While it was known that struvite calculi form in the presence of urease-producing bacteria (*Proteus mirabilis*, *Klebsiella pneumoniae*, *Staphylococcus aureus*, *Pseudomonas aeruginosa*, etc.) [78], it is now necessary to consider that urinary bacteria are also involved in the formation of calcium oxalate stones, with the exact mechanism yet to be elucidated. The urinary microbiota could act by altering the urine pH, producing metabolites that influence the solubility of minerals in the urine, interacting with substances like citrate that inhibit stone formation, increasing oxalate concentration, or serving as nuclei for mineral crystallization [22,31,64,79,80,81]. 

### 4.5. Advantages of This Animal Experimental Model

The 18 cats studied demonstrate the benefits of an animal experimental model, as the individuals studied were of the same breed, were sexually neutral from a young age, were of the same age (about 10 years), came from the same colony, had the same medical history, lived in the same environment (for 9 years), all received the same standard diet every day, had never been given antibiotics, and they all received the same dose of antibiotic (with a bioavailability of 99% and a duration of action of at least 14 days). The cats in the study were not in renal failure, so the factors of microbiota variation associated with renal failure failure [80,82] are not taken into account in this study.

Regarding urine collection, this study goes beyond the recommendations proposed by MICROCOSM using cystocentesis, the method of choice for urine collection in cats. It avoids the risk of contamination with urethral bacteria [75]. This technique is easy, safe, as well tolerated as a subcutaneous injection, and does not require ultrasound guidance for cats with a palpable bladder.

Although there are two male cats in the group of healthy cats and 16 females distributed between the two groups, none of the cats in the study has ever experienced hormonal influence as all cats were sterilized before puberty. Therefore, the composition of their microbiota has not been influenced by the presence of sex hormones. Anatomical differences between male and female cats only concern the lower urinary tract, not the upper urinary tract. Several studies conducted in mice and in humans have not shown an influence of sex on microbiota composition [83]. In the studies conducted in humans with kidney stones, no distinction was made between men and women; our cats have the advantage of all having the same sexual status—they have all been sexually neutered since birth.

The exact composition of the kidney stones was only identified for the three cats that died out of the nine cats with lithiasis; as expected, for these three cats, the body of the stones consisted of 100% calcium oxalate monohydrate. The oxalocalcic nature of the other six stones was assumed on a probabilistic basis knowing that 98% of upper urinary tract stones are calcium oxalate stones in cats [7,9] and given that struvite stones have a distinct radiological appearance; they are less radiodense, larger, smoother, and sometimes pyramid-shaped. Thus, there was a very low risk that one of the six cats had stones that are not calcium oxalate. Some studies in human medicine did not distinguish between the types of stones [19,32], while calcium oxalate stones represent only 80% of all kidney stones in humans.

The veterinary studies [17,27,39] enrolled dogs with calcium oxalate stones belonging to owners, so dietary and environmental factors were highly variable. In the eight previous studies conducted in human medicine, variables like diet, lifestyle, diseases, medications, and geographical region were not taken into account, yet, they are significant factors leading to individual variations in microbiota composition [84]. The possibility of erasing certain environmental factors makes the cat raised under experimental conditions an excellent study model for human urolithiasis.

The present study paves the way for a more comprehensive analysis of the composition of the intestinal and urinary microbiota through global metagenomics (shotgun) and an analysis of its metabolic by-products [80], not merely observing the presence or absence of individual bacterial species. 

### 4.6. Limitation of This Study

Due to its observational design, this study cannot establish a causal relationship between the composition of intestinal and urinary microbiota and the occurrence of kidney stones [85]; it is possible that the dysbiosis observed is a result of the presence of stones rather than a cause.

### 4.7. A Hypothesis on the Origin of the Epidemic of Kidney Stones in Cats

None of the cats in this study that spontaneously developed lithiasis had ever received antibiotics during their lifetime; this is also the case for many cats that spontaneously develop kidney stones. Therefore, antibiotic therapy is not the sole factor responsible for the lithiasis epidemic observed in cats. Diet is the factor that most affects the composition of the microbiota. The event that most likely contributed to the increase in the incidence of kidney stones in cats was the trend, initiated in the late 1980s, to manufacture foods that acidify the urine to prevent struvite stones in the lower urinary tract, which were very common at the time. Alkaline urine is more conducive to the formation of calcium oxalate stones, and thus to the formation of kidney stones. A second dietary risk factor is that modern cats are more often fed dry food (kibble), which makes the urine more concentrated and thus facilitates the hypersaturation of ions. 

Despite the dietary measures taken by pet food manufacturers in recent years, the epidemic of kidney stones in cats has not been significantly curbed, that is why we also suspect a hygiene hypothesis that the disturbance of the intestinal microbiota could results from the following set of factors inherent to the lifestyle of cats in our modern societies:

- many cats living in urban environments go out a few times or not at all, so they have fewer opportunities to seed their digestive tract in their youth with the microbiota of their prey or that of their congeners,
- they eat only industrial foods that are almost sterilized for preservation concerns,
- they are more medicalized so they receive antibiotics more often than in the past.

The dysbiosis associated with the new lifestyle of cats living in modern societies could be the origin of feline diseases whose incidence is on the rise, including obesity, diabetes, chronic inflammatory bowel diseases, hypertension and now nephrolithiasis, as observed in humans. This phenomenon is transgenerational because a female cat with a less diverse microbiota will give birth to kittens whose microbiota will also have a low diversity.

## 5. Conclusions

Renal lithiasis has become a major concern in feline medicine. Understanding the role of the intestinal and urinary microbiota in the formation of kidney stones in cats is of interest for the development of new therapeutic and preventive approaches: strategies based on the modulation of the intestinal microbiota, such as dietary changes, the use of probiotics or prebiotics, fecal microbiota transplantation, or even urinary microbiota transplantation, could be considered to prevent the formation of renal calculi in cats.

## Data Availability

All data are available on request.

## Supplementary Materials

The following supporting information can be downloaded at: https://www.mdpi.com/article/10.3390/microorganisms12061098/s1. The composition of the food can be found on the website: https://www.royalcanin.com/au/cats/products/vet-products/mature-consult-2724 (accessed on 23 April 2024).
