# Supplementary material for: Gut and Urinary Microbiota in Cats with Kidney Stones
Source: Microorganisms. 2024 May 29;12(6):1098. doi: 10.3390/microorganisms12061098 (PMC11205531; doi:10.3390/microorganisms12061098)
Supplement: Supplementary file 1 [file microorganisms-12-01098-s001.zip › microorganisms-3003030-supplementary.pdf]

Diet composition of the Mature Consult Balance S/O, Royal Canin®
https://www.royalcanin.com/au/cats/products/vet-products/mature-consult-2724

Composition: Dehydrated poultry protein, maize, wheat gluten*, rice, wheat, maize gluten, vegetable fibres, hydrolysed animal proteins, minerals, chicory pulp, animal fats, fish oil, soya oil, dried tomato pulp (source of lycopene) (0.80%), psyllium husks and seeds, fructo-oligo-saccharides, hydrolysed yeast (source of manno-oligo-saccharides), glucosamine from fermentation (0.048%), marigold meal (source of lutein), hydrolysed cartilage (source of chondroitin) (0.00048%).

Additives (per kg): Nutritional additives: Vitamin A: 20500 IU, Vitamin D3: 755 IU, Iron: 35 mg, Iodine: 3.5 mg, Copper 11 mg, Manganese: 45 mg, Zinc: 131 mg, Selenium: 0.06 mg - Preservatives - Antioxidants.

Analytical constituents: Protein: 36.0% - Fat content: 10.0% - Crude ash: 7.1% - Crude fibres: 5.4% - Phosphorus: 0.8% Calcium: 1.0% - Sodium: 0.7% - Magnesium: 0.07% - Potassium: 0.7% - Chloride: 1.14% - Omega-3-fatty acids: 0.71%
